# Medical Items and News of Interest to the Physician

**Published:** 1874-07

**Authors:** 


### Medical Items and News,

### For the Use of Physicians.

CULLED FROM THE STANDARD MEDICAL JOURNALS OF THE WORLD.

NOTE.—These formulæ are intended only for the regular practitioner of medicine, and should be employed only by him, or under his immediate supervision.

**Night-Sweats of Phthisis.**

House-Physician Smith remarked that two-grain pills of oxide or zinc, t. i. d., has answered a better purpose in his division for controling this symptom than any remedy that had been employed.—*Ex.*

---

**Depilatory Mixture.**

A new depilatory mixture is recommended by Prof. Boettger, consisting of one part of crystallized sulphydrate of sodium, and three parts of prepared chalk. Mixed with water, and applied to the skin, it effects the easy removal of hair.—*Med. and Surg. Reporter.*

---

**Chilblains and Coryza.**

For chilblains and chaps: Alcohol, 100 parts; glycerine, twenty-five parts; and carbolic acid, one part. Powdered camphor sprinkled with tincture of iodine and inhaled by the nostrils, constitutes one of the most prompt and certain of remedies in coryza, or "cold in the head."—*Med. Times & Gazette.*

---

**Syr. Morphæ Comp.**—(Jackson's Cough Syrup.)

℞

Ext. Ipecac. fluid f ʒ ss.

" Senegæ, " f ʒ iij.

" Rhei, " f ʒ iv.

Syr. Simplicis, f ℥ xxxj.

Morphiæ muriatis grs. viij.

Ol. Sassafras, gtt. xxxij.

M. Ft. Mist.

---

**Neuralgia.**

Dr. Edward C. Hurse has employed with success, in a large number of cases of neuralgia the following combination of ergotine with the phosphate of zinc:

℞

Zinci phosphidi, ʒ j;

Ergotin, gr. v.

In piulas No. 60 dividend. Sig. One pill to be taken after each meal.—*Richmond & Louisville Med. Journal.*

**Sugar and Magnesia an Antidote to Arsenic.**

THE *Mouvement Medical* relates various experiments conducted by Mr. Carl, with the result of showing that sugar mixed with magnesia may serve as an antidote in cases of poisoning by arsenious acid, in which cases, too, the internal use of the hydrated magnesia is most valuable.

---

**Incontinence of Urine.**

Dr. Thos. Kennard, of New York, uses the following ointment in the treatment of this disease:

℞

Sul. atropia, gr. x;
Veratria, gr. x;
Hog's lard, ʒ xij. M.

By rubbing the perineum three times daily, with the ointment, in three cases of paralysis accompanied by incontinence of urine, Dr. Kennard obtained a complete recovery at the end of a few days.

---

**Etheral Tincture of Phosphorus.**

Powered phosphorus 1 part.
Ether, 50 parts.

Macerate for a month with occasional shaking. Decant into small vials covered with black paper.

It has been objected, justly we believe, that all tinctures of phosphorus, ethereal or alcoholic, are very unreliable, on account of the rapid oxidation of phosphorus when in such a state. The strength of all the liquid preparations of phosphorus speedily decreases, in spite of all precautions.

---

**Treatment of Cholera.**

Dr. J. H. Parrish, of Gallatin, Tenn., writes the *Med. and Surg. Reporter*, as follows:

I was in the midst of cholera last summer. My treatment was in the main satisfactory. I gave calomel and opium freely, and had much satisfaction from the following:

℞

Camphor water, f. ℥ vj;
Gum arabic, grs. cxx;
Oil mint, gtt. xxiv;
Creosote, gtt. xxxxj.

Make an emulsion. Dose: One tablespoonful every one, two or three hours. After having given calomel and opium, the above emulsion rarely failed to arrest vomiting and purging. I also used, very freely, enemas of benne water.

**Tasteless Cod Liver Oil.**

℞

Cod liver oil, ℨ ij;

Spts. lavender, comp., ℨ j;

Brandy ℨ j. M.

The house Surgeon said the patients begged for it.—*Rosevelt Hosp. Rep.*

---

**Flatus.**

℞

Pulv. camphor,
" capsicum,
" ginger, āā gr. j. M.

Divide into six pills. Sig. One p. r. n. Said to afford immediate relief.—*Rosevelt Hosp. Rep.*

---

**Mouth Wash.**

A very good gargle and mouth wash may be made by taking—

℞

Fld. ext. rhus glab, f. ℨ iv ;

Potass. chlorate, ℨ ij ;

Glycerine, pure, ℨ iv;

Water, ℥ vij.

Filter.

—*Journal Pharmacy.*

---

**Gonorrhœa.**

Injections of silicate of soda have been employed in the treatment of this affection in both acute and chronic stages.

℞

Silicate of soda, grs. xx ;
Aqua, ℥ viij.

The injections were given three times a day. So far as employed, the results obtained were eminently satisfactory. No other treatment was combined with it.—*Rosevelt Hospital Reports.*

---

**Ulcers.**

This class of cases, "old ulcers," are quite uniformly dressed with Labarraque's solution (liq. sodæ chlorinatæ) until the sore becomes surgically clean. The solution is be diluted with water, according to circumstances. If then the granulations have a healthy appearance, the ulcer is strapped, and the limb bandaged. If the granulations become flabby and inactive, a dressing of balsam of Peru is applied, and over that straps and bandage. Grafting is resorted to in certain cases, when the ulcer is of some size, and, in this manner, a certain proportion are made to heal very rapidly.—*Rosevelt Hosp. Rep.*

**Burns.**

For this class of difficulties whitelead paint seems to meet the indications of treatment as satisfactorily as any material which has been employed. This is interesting, from the fact that almost every plan of treatment which has ever been devised has been employed, and the leaning is towards the paint dressing. Mix as for painting, except considerably thicker, and apply with a brush.

---

**Tincture of Guaiacum for Hoarseness.**

On the authority of a very prominent physician of Philadelphia, in hoarseness or loss of voice, arising from thickening of the vocal cords and adjacent membrane, the ammoniated tincture of guaiacum is often a very efficacious remedy. It may be appropriately mixed with equal parts of syrup of seneca, and a teaspoonful of the mixture given to an adult two or three times a day.—*American Practitioner.*

---

**Cholera Mixture.**

The following is published as the "Cholera Mixture of the British Army :"—

Oil of anise seed,
" cajeput,
" juniper, of each 3 drachms.
Ether 8 "
Liquor acid of Hallar 1 drachm.
Tincture of cinnamon 4 ounces. M.

Dose, ten drops every quarter of an hour, in a tablespoonful of water.

"Liquor acid of Hallar" consists of one part (by weight) of concentrated sulphuric acid to three of alcohol.

---

**Ascaridies.**

According to Szerleki, of Mulhouse, ascarides are effectually removed by injections, daily for a few days, of six to eight drachms of pure cod-liver oil.—*Boston Med. Journal.*

We know this remedy to be effectual, if continued for three months. The worm deposits immense numbers of eggs which come to maturity, producing the little worm which is the cause of the terrible itching, in from four to six weeks. Hence the periodical attacks from these little pests The oil should therefore be continued long enough to insure the complete destruction of the different broods, which succeed each other in about the time indicated.—Ed. Bistoury.]

**Expectorant Mixture.**

The *Medical Record* states that an expectorant mixture much used in the New York Charity Hospital in cases of chronic bronchitis, and with very good results, is the following :

℞

Ammon. muriat.,

Liq. morph. sulph. (Mag.) āā., ʒ j;

Syr. tolu,

Syr. scillæ co., āā., ℥ j. M.

S. ʒ j. ter in die.

---

**Liniment for Acute Articular Rheumatism.**

The following is used in the same institution, as an application for the joints in this form of rheumatism :

℞

Tr. opii, ℥ j;
Spts. chloroform, ℥ iss;
Lin. saponis, ad O j. M.

This liniment is applied freely over the joints, and immediately covered with cotton and oil-silk. The relief from pain afforded by this application has been very gratifying to all the rheumatic patients. The general treatment is alkaline.

---

**Carbolic Acid in Tapeworm.**

In a recent issue of your valuable journal I noticed a letter from Dr. Bills, of the army, recommending the use of carbolic acid in the treatment of tapeworm.

Having a case on hand at the time which had resisted the use of oil of turpentine and bepo for a month or six weeks, I immediately administered to him the following prescription : ℞ Acid. carbolic cryst., gr. ij.; glycyr. pulv., gr. iv. Ft. pil. No. 2. To be taken every three hours, beginning at 9 A. M. and continued until 9 P. M. This, followed in the morning by jalap and rhubarb ten grains each. On the first day a few joints were passed, on the second a great number, and on the third the head and narrow portion came away, with a great number of small sections from one to three inches long. On the fourth day, treatment being kept up without further result, the medicine was discontinued.

The only sensation created in the stomach by the administration of such large quantities of the drug was a slight burning, not sufficient to inconvenience the patient. I look upon this as a perfect cure, and therefore report the case to you. — S. AUSTINE BROWN, M. D., in *The Medical Record.*

**Valerian in Diabetes.**

The assertion is made by Dr. Bouchard, that the extract of valerian is a powerful agent in diminishing the elimination of urea, and the waste of tissue seen in cases of diabetes. If azoturia complicates glycosuria, the urea diminishes more and more.

He adds a curious fact, based on long practice among the Indians of Lower California. The warriors, before entering on an expedition, go through a course of valerian regimen for a month, and render themselves in a fatigue-supporting condition.—*Med. & Surg. Reporter.*

---

**Gonorrhœa, Gleet, Etc.**

We have recently known a number of very obstinate cases of gleet relieved by the introduction of a catheter, smeared with mild zinc ointment, once or twice per day. Many recent cases of gonorrhœa are much relieved by the same means, with the addition of a little carbolic acid, sulphate of zinc or nitrate of silver. An injection, containing about 2 grs. of sulphate of zinc to the ounce of water, and the whole made thick as cream, with finely-powdered goldenseal (Hydrastis Canadensis), is deemed worth from $500 to $1,000 by those who have been very speedily cured by it. At least, such is their verbal estimate of its value. It is thrown into the uretha, and allowed to remain as long as it will.—*Med. Times.—Canada Lancet.*

---

**Remedy for Toothache.**

Having spent a number of years of my professional life in the country, where no dentist was near, and therefore being often called to prescribe for toothache, I tried many combinations of drugs, and many published formulas, with more or less satisfaction to myself and patients. But several years since I hit upon the following combination, which I have found better than I have ever used, and it will give relief in almost all cases where such a remedy is applicable :

℞. Carbolic acid, saturated solution ; hydrate of chloral, saturated solution ; camphorated tinct. of opium ; fluid extract of aconite, of each an ounce ; oil of peppermint, ½ oz. Mix. Apply by saturating a pledget of cotton (or preferably a small piece of sponge), and pack closely into the cavity of the decayed tooth.—Dr. Q. C. SMITH, in *Pacific Med. & Surg. Journal.*

**Ergotin in Prolapsus Ani.**

The eminent Surgeon, Von Langenbeck, of Berlin, announces that he has lately been treating prolapsus ani, "with astonishing success," by hypodermic injections of a solution of ergotin (five to fifteen parts to one hundred of distilled water). He replaces the bowel, and inserting the point of the syringe about three centimetres in depth in the cellular tissue, throws in from one to two grains of ergotin. This should be repeated every three or four days for three or four weeks, any hard fecal masses in the bowels being first removed by a simple injection. As a means of treating a most obstinate and troublesome complaint, this method, sanctioned by so eminent a name, deserves careful repetition.

---

**Abortive Treatment of Coryza.**

Dr. Prout, of Brooklyn (*Med. Record*), recommends the tincture ferri chlor., in large doses, as a valuable remedy in the early stage of coryza. As soon as possible after one is seized with the cold in the head, give half a drachm of the tincture, in glycerine and water. In half an hour the symptoms are readily ameliorated, and it may not be necessary to repeat the medicine. But if the improvement be but temporary, the dose must be repeated. It may be necessary to give two or three doses. Dr. Prout does not claim that the treatment is always successful, but mostly so, when instituted as speedily as possible after the attack. No unpleasant effect follows, except a slight and transient feeling of discomfort in the bowels. There is a slight diuretic action.

---

**Treatment of Angina Pectoris.**

Dr. Hueber, Lioland, believes that the only permanent relief for Angina Pectoris is to be obtained through the galvanic battery. In most cases of this kind, where auscultation and percussion reveal no material change in the heart, the phenomena are so characteristic of nervous affections that the disease should be regarded as a nervous one. The cardiac plexus is the seat of the difficulty, and hyperæsthesia provokes the alarming train of symptoms.

In an obstinate case which came under his care, Dr. Hueber employed a number of remedies without success, and finally had recourse to the galvanic current.

He employed Stohrer's battery with thirty elements. The cord was found intact, but on applying the current over the sympathetic, only four elements were borne on the left side, and six on the right. After the first sitting the attacks ceased, and never returned. The patient was soon able to bear six elements on the left side, and eight on the right; and, finally, an equal number could be applied on both sides. Thirteen applications in all were made. It is proper to add that the valerianate quinine was continued without interruption at the same time, though from previous experiences it was not believed to have effected the permanent cure. —*Deutche Archiv f. Klin. Med.*, *Allg. Wien. Med. Ztg.*, 2, 1874.

---

**Treatment of Gastralgia.**

Dr. Joulin, in *La France Medicale*, commends the following treatment for gastralgia: 1. A poultice of ice for ten minutes, morning and evening, to the pit of the stomach. 2. A mustard plaster to the same spot immediately on removing the ice poultice, to be kept on as long as possible. 3. Pounded ice, to be taken morning and evening, a tablespoonful every five minutes for one hour. 4. A mustard bath with two pounds of mustard three times a week. The ice of the poultice is to be chopped up into small bits and enclosed in an india-rubber bag. The ice taken internally must be pounded with sugar. It must be swallowed down suddenly, and not kept in the mouth, as it would lose its coldness. If a tablespoonful would be too much at a time, several teaspoonfuls may be taken instead. The mustard bath Dr. Joulin regards as a powerful tonic.

---

**A Case of Opium Cure.**

*The Druggist*, of London, says that a young lady who had been long accustomed to the use of opium applied to an eminent physician to make hypodermic injections of morphia. He commenced by making the injections as desired, of morphia and water; by degrees the quantity of morphia was lessened without her knowledge, until within a few days nothing but pure water was injected; after each injection she would lapse into a quiet sleep, in the same manner as she had been accustomed to do under the actual use of morphia. This treatment was continued for several months, during which time tonics had been used, to strengthen the system and bring about a healthy condition after being so long under the influence of opium. When he considered it safe to do so, he told her plainly that she had not taken a particle of morphia for several months, and was entirely free from its influence; this statement of course was received with intense surprise, as well as unbounded joy. The lady is to-day entirely free from any desire for opium.

---

**Climate in Consumption.**

We reprint the following sensible and interesting article, taken from the *Medical Record*, and by them gleaned from clinical lectures in the New York Hospitals. It is worthy the consideration of all practicing physicians, containing as it does, more practical, common sense than is usually afforded in articles upon the subject.—Ed. Bistoury.

"First, a proper atmosphere should be given the patient to live in. An air should be attained, if possible, of such a nature that it can be breathed for the greater part of the twenty-four hours. Second, give the patient the benefit of sunlight. Third, avoid an atmosphere loaded with dust. Fourth, exercise without producing fatigue. The climate should be selected, if change of climate is to be made, in which the patient can be the most comfortable for the greater part of the 24 hours, for that is the climate which is to prove the most beneficial. The temperature and locality are generally secondary considerations. As a rule a low and damp location is injurious.

If the patient cannot be exposed to outdoor air without feeling chilled, and is necesitated to sit by a fire when he returns, for the sake of again feeling comfortable, it is much better for him to remain in the house and make an atmosphere for himself by artificial means, and then take regularly such light gymnastic exercise as will keep the body in the best possible condition. If change of location is to be made, the patient should be sent to some place where he will have a good time. Never send a patient where he will be surrounded by depressing and unhappy influences. Never send a patient in the advanced stage of consumption away from home, unless he is so situated that he can take his time and all its comforts with him. Never send a patient with consumption to the sea shore, unless the experience of the individual has proved such an atmosphere beneficial. In general it does harm. Some favor long sea voyages in the early stages. Especially avoid the habit of sending every patient to the same place. In case the patient enjoys hunting and fishing, if he can be sent to a locality where he can have his favorite sports, it will prove an additional item in his favor. The man who is fond of these sports is much more likely to get well of his phthisis. If these external conditions can be controlled, no great amount of anxiety, as a rule, need be manifested with regard to the *cough*. It is the cough generally that most attracts the attention of the patient. The great object to be held in view is to raise the health of the patient to a level above that in which phthisis progresses. The power of drugs to accomplish this is very doubtful. Alcohol is of doubtful efficacy. If used at all, it should be used with a view of assisting digestion; and the article selected should be that most agreeable to the stomach. Air, free from moisture and dust, food and exercise, are the things to be relied upon in the general treatment of this disease. There are certain effects of the disease which the physician will be called upon to counteract, and for the relief of these he may be obliged to resort to some theraputical measures. It is to these conditions and measures that attention is now especially directed."

---

**Hypodermic Injection of Carbolic Acid in Erysipelas.**

Dr. Aufrecht, of Magdeburg, having last year lost four patients of advanced age, who were attacked by erysipelas of the extremities after injury, determined to try the effect of carbolic acid, and in a short paper in the *Centralblatt* for February 21, he communicates the results which he obtained in two cases. If (he observes) it be true that erysipelas in such cases as these arises from the penetration of organisms into the subcutaneous tissue, and their multiplication there, and if carbolic acid possess the power of destroying such organisms, or of impeding their injurious influence, this substance should be able to prevent the spreading of the erysipelas, and to a certain extent diminish its danger. In order to ascertain whether carbolic acid may be hypodermically employed without any ill consequence, he experimented upon himself with a 1 per cent. solution, of which he threw in six decigrammes at a time—*i.e.*, the amount contained in an ordinary Pravaz's syringe. Neither local nor general ill-effect resulted. Since then he has employed the injection in two cases—the one a woman aged fifty-six, with erysipelas of the forearm and the hand, arising from a slight abrasion; and the other a man, aged eighty-two, with erysipelas of the thigh, following slight ulceration of a cicatrix. In the first case five injections were employed during three successive days, and in the second four injections within two days. The injections were thrown into the sound subcutaneous tissue, just beyond the margin of the erysipelas, as it advanced toward the trunk. Its progress was at once arrested in the direction where the injections were made, the injection being repeated in consequence of some insular erysipelas appearing beyond the first injection-points. More remarkable still than this limitation of the erysipelas was the decided influence of the injections in diminishing the febrile action and the frequency of the pulse, and in inducing a general improvement in the patients' conditions. Convalescence was quite satisfactory in both patients.—*London Medical Times and Gazette.*

---

**Spinal Anæmia.**

On examination of the spine, tenderness on pressure was found from the third to the tenth dorsal vertebra, most marked over the eighth and ninth; and there was tenderness also over the third and fourth lumbar vertebræ. Slight pain in the cord, chiefly in the upper dorsal region, was developed by percussion. The case was diagnosed to be one of anæmia of the posterior columns of the spinal cord.

Blisters over the seats of tenderness were ordered to be applied, and repeated as soon as possible. The following prescription was given:

℞
Strychniæ sulphat., gr. j;
Ferri pyrophosph.,
Quiniæ sulph., āā ʒ ss;
Acid. phosph. dilut.,
Syrupi zingiberis, āā f ℥ ij. M.

S. A teaspoonful to be taken three times daily, immediately after each meal.

The induced current was applied twice weekly to the muscles of the body and extremity. Exercise in the open air, and good nutritious diet, were also enjoined.

At the time of making this report the patient has been three weeks under the treatment just given, the blisters having been twice repeated. The palpitations, neuralgic and other pains, flatulence, acidity, nausea, and nocturnal emissions have ceased to annoy him. He experiences very little uneasiness in the head, and can walk a long distance without difficulty. He expresses himself as feeling better than he has done before for nearly three years. The same treatment will be continued.—*Hospital Report in the Philadelphia Med. Times.*

---

**Local Treatment of Pulmonary Cavities by Injections Through the Chest-wall.**

We have long been of the opinion, abundantly fortified by our experience, that the theraputics of pulmonary diseases, heretofore employed, has been worse that useless. We have been hopeful that some daring investigator would attempt to treat pulmonary cavities by injecting remedies directly into the diseased tissues, bringing the medicine in direct contact with the diseased surfaces. This at last has been accomplished, and we are hopeful of the best of results. The experiments were made by means of Dieulafoy's aspirator, in the hands of Prof. Pepper, and the following is taken from his report.—Ed. Bistoury.]:

"On February 20th, the No. 1 needle of Dieulafoy's aspirator, with the syringe attached, was introduced to the depth of about one inch in the second interspace on a line with the right nipple. He complained merely of a slight sensation of tingling and numbness, extending down the right arm. There was no cough excited and not a drop of blood followed the puncture.

"February 24th—The same needle was introduced at the point of the original puncture to the depth of one and seven-eighths inch, and was followed by an escape into the vacuum of a few drops of offensive pus. The needle was very tightly bound by the tissues through which it passed, but some motion could be imparted to its free end, indicating that it was in a free space. Three or four minims of a mixture composed of liq. iodinii, comp., gtt. ij to f. ʒ j of tepid water were then injected through the canula by a hypodermic syringe.

"The operation was followed by a loose, rattling cough, and the expectoration of about three fluid drachms of fresh, frothy blood.

He was immediately put to bed, and the cough and hemorrhage soon stopped. His temperature in the evening and the following morning was only 99° F.

"On February 28th, six minims of iodine solution, of double the former strength, were injected at the same point and in the same manner.

"On March 5th, ten minims were injected at a point one-eighth of an inch nearer the sternum.

"March 8th it is recorded: The patient is brighter and more cheerful than for months past. There is no hectic irritation, the temperature never rising above 99° or 99.8°. The pulse ranges about 84. His breathing is so much relieved that he has walked up three flights of stairs without much dyspnœa. The cough is but little troublesome and only a few white, frothy sputs are raised. Auscultation shows that many of the rales formerly heard over the right apex have disappeared. There has also been some increase in flesh. All internal medication has been discontinued."

---

**Vomiting of Pregnancy.**

Dr. D. B. Simmons, of Yokahoma, recommends the administration of hydro-chloral *per rectum* to allay the persistent harrassing vomiting of pregnancy. He reports four cases, in which, after the failure of the usual remedies, such as oxalate of cerium, hydrocyanic acid, morphia, &c., the distressing symptoms were made to speedily disappear by injection of 30 grains of hydro-chloral into the rectum morning and night.—*Medical Record.*

---

**Vomiting After Meals.**

This symptom is quite common. A wineglass of champagne, taken during meals, will sometimes arrest it at once. Hydrocyanic acid dil., in doses from ♏ iij, gradually increased to ♏ vij, administered just before each meal, is another remedy. The condition is ordinarily one of irritability, and if the acid can be so as to produce marked sedative effects, it may be of great service.

Pepsin and muriatic acid, compressed soda pills (English preparation, grs. viij, each), one before each meal. *Raw beef scraped or cut fine and seasoned.* Let the patient take teaspoonful or half teaspoonful doses every hour or two, or oftener, and subsist upon this alone for a few days. The effect is very beneficial in many cases.

